# Cardiac puncture blood collection as a practical and biosecure method for post-mortem pathogen detection in pigs

**DOI:** 10.3389/fvets.2025.1741832

**Published:** 2026-01-12

**Authors:** Claudio Marcello Melini, Mariana Kikuti, Xiaomei Yue, Cesar A. Corzo

**Affiliations:** Department of Veterinary Population Medicine, University of Minnesota, Saint Paul, MN, United States

**Keywords:** alternative specimen, intracardiac blood, pathogen detection, post-mortem, porcine reproductive and respiratory syndrome virus (PRRS)

## Abstract

Live pig specimen collection can be time-consuming as it may require physical restraining. Non-invasive methods for live animals that don't involve blood spillage are available but some may present limitations of application or pathogen detection. Alternative methods focused on the mortality are also available but may derive in blood spillage. As biocontainment and bioexclusion are of concern, especially for pathogens such as the ones that cause classical swine fever and African swine fever, alternative methods can be applied. This article presents the results and opinions of the use cardiac puncture (CP) as an alternative of specimen collection from dead pigs. This method was used in two separate studies involving porcine reproductive and respiratory virus detection from blood of dead pigs of different ages. Blood was successfully obtained from 286 suckling and growing pigs, that were euthanized or died during outbreaks of the tested virus. While only 273 samples could be tested due to the other 13 not yielding enough sera of having inhibitors, of those 95% had positive detections for the virus. This method was not only feasible for obtaining blood and test the sera, but also avoided environmental contamination with blood, offering an alternative tool for collection during outbreak investigations.

## Introduction

Successful diagnostic specimen collection from live pigs requires time and skills. Non-invasive pre-mortem sample collection methods using swabs (i.e., oral, nasal, rectal, genital tract), wipes (i.e., udder, snout), semen, tonsil oral scraping, and oral fluids require a short interaction with the pig and for most, lack of physical restraining (1–3). Blood sampling is considered invasive, and in pigs there is the need for physical restrain for the safety of the animal and sample collector (2). Alternative sampling can be conducted in breeding herds by using the recovered serosanguinous fluid from testicles and tails after piglet castration and tail docking (processing fluids) (4–7), but in countries where these activities are forbidden because of animal welfare (8) or in grow-finishing herds were these practices are not conducted, post-mortem sampling can be an alternative for specimen collection during both endemic and foreign animal disease (FAD) infectious disease outbreak diagnostic investigations. Post-mortem sampling collection it is usually

performed through a necropsy to assess macroscopic lesions and collect specimens for different types of testing (i.e., histology, molecular diagnosis, bacterial and viral isolation). Blood spillage during necropsy is common, as the incisions made to reach specific organs may cut blood vessels. In swine farms, necropsies can be performed by workers or veterinarians, in the case of the former they can request for support to identify lesions by a trained professional using telemedicine, but this can have its own limitations (9). Routine specimen collection and diagnosis in swine farms may be limited to its easiness of obtention, availability, staff qualification, and suspected agent. But ultimately, it should be driven by a well-defined diagnostic question (10). In the case of a disease such as porcine reproductive and respiratory syndrome (PRRS) which is endemic in most pig producing countries, blood contaminated (11) surfaces are of concern as these can act as fomites. However, in the case of an FAD such as African swine fever virus (ASFV), it has been reported that the virus can remain viable in putrefied blood for 15 weeks at room temperature (12), meaning environmental contamination with blood during these investigations represent an important dissemination risk which during the early stages of the epidemic needs to be mitigated. Even though blood is a preferred diagnostic specimen given its proved value (13), tissue collection during ASFV and classical swine fever virus (14) investigations is common. Minimal invasive post-mortem specimen collection alternatives are needed. Cardiac puncture (CP) blood collection can be easily obtained as an alternative for pathogen detection, as it is a method known anecdotally amongst veterinarians. Although, it has been described *in vivo* for swine (15) and poultry (16) it is not frequently used in swine practice. In this perspective article, we share our experience using post-mortem CP in two separate studies for the detection of PRRS virus (PRRSV) while comparing it to other commonly used specimens.

## Post-mortem intracardiac blood collection

**Figure 1:**
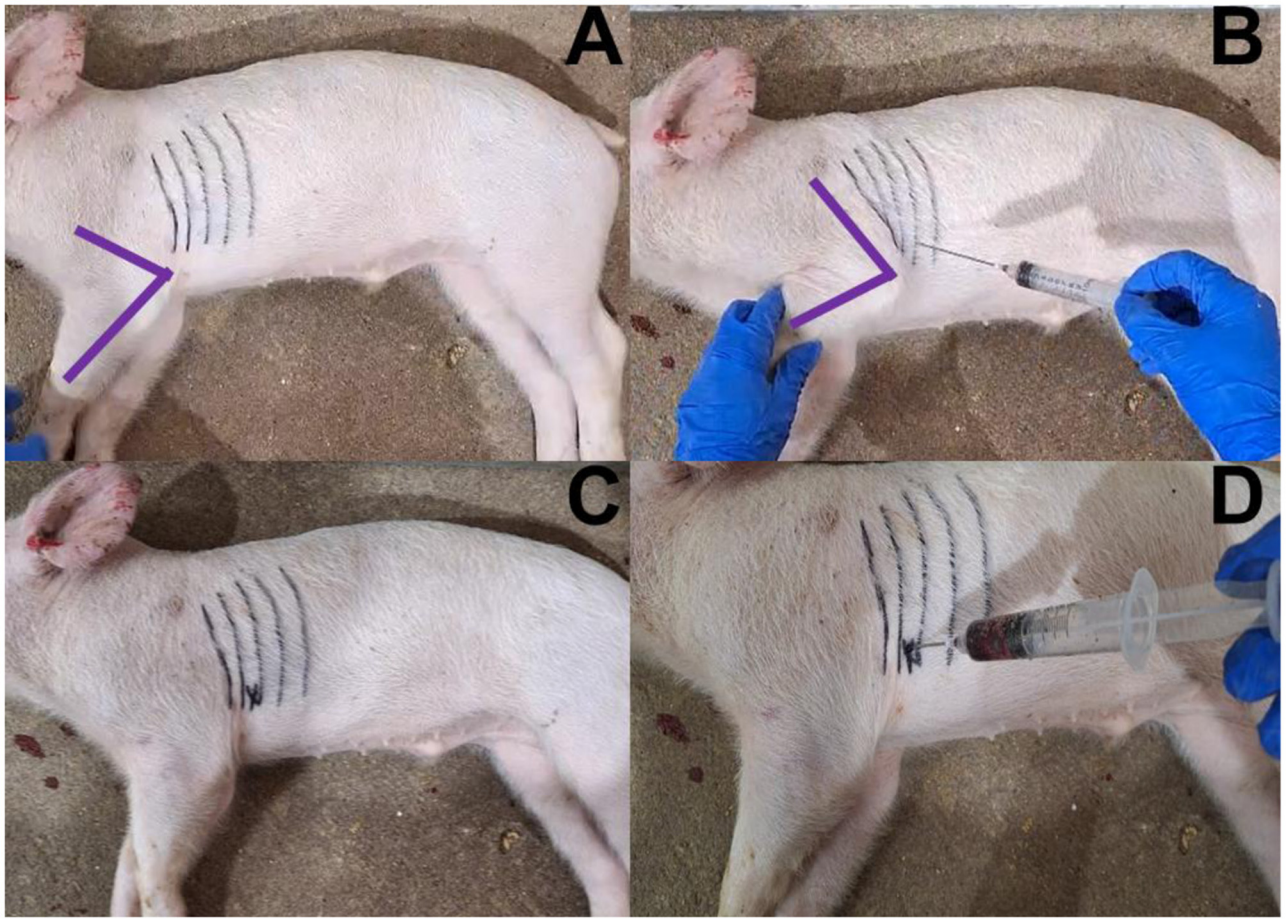
Photos of cardiac puncture blood collection sequence from a dead weaned pig. **(A)** Placement of pig on right lateral recumbency (forelimb is depicted in purple, ribs are marked in black); **(B)** Identification of puncture area (intersection between olecranon and intercostal space); **(C)** Marked puncture area with an X after releasing the forelimb; and **(D)** Insertion of needle in puncture area, retraction of the syringe's plunger, and collection of intracardiac blood.

**Figure 2:**
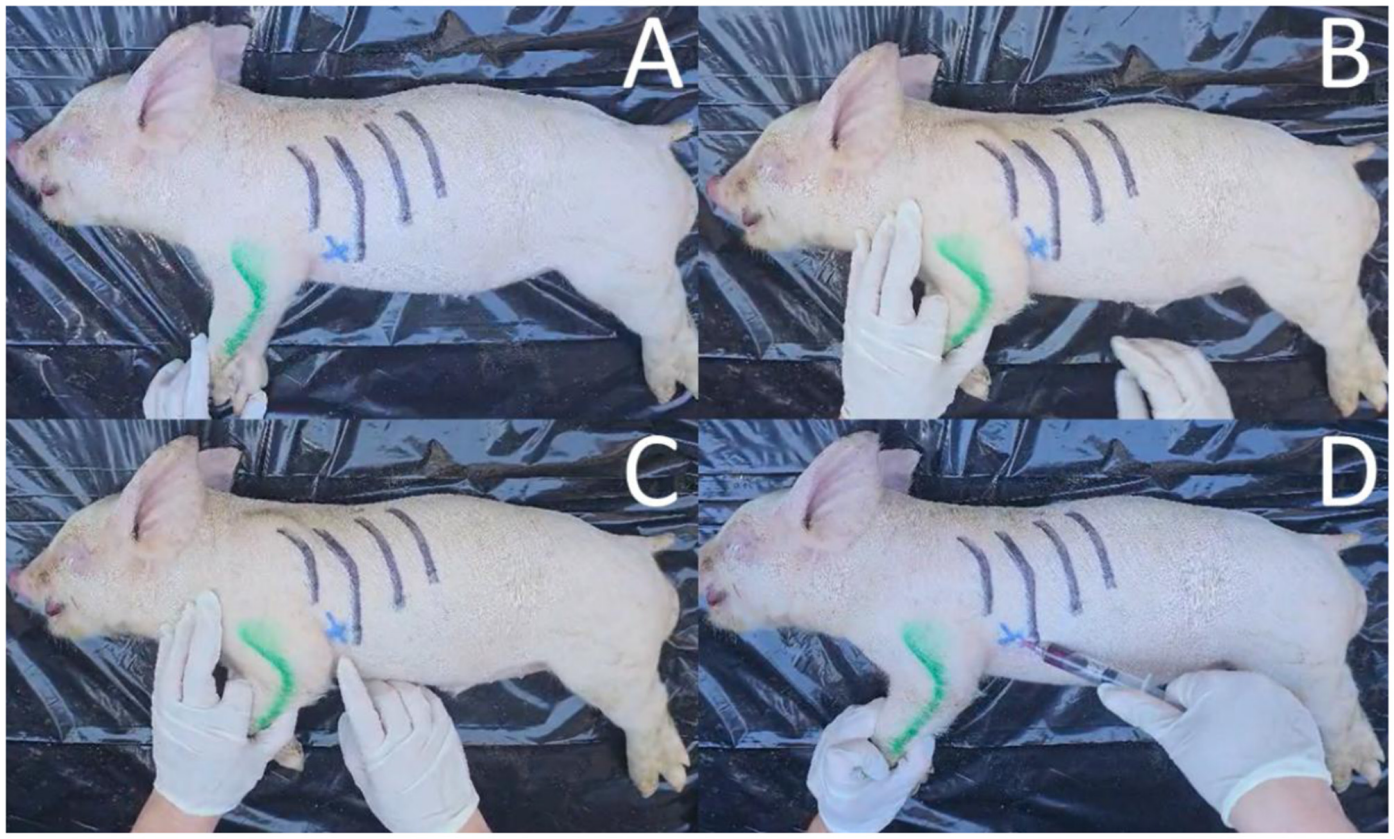
Photos of cardiac puncture blood collection sequence from a dead weaned pig. **(A)** Placement of pig on right lateral recumbency (forelimb is depicted in green, ribs are marked in black); **(B)** Identification of puncture area (intersection between olecranon and intercostal space); **(C)** Marked puncture area with an X; and **(D)** Insertion of needle in puncture area, retraction of the syringe's plunger, and collection of intracardiac blood.

From both studies (17, 18), blood samples were collected at five Midwestern United States farms in which three were breed-to-wean and two were growing pig farms undergoing a PRRSV outbreak. The age of inclusion and sample size for each study was determined based on the objectives of each of the studies, such as the detection of at least one PRRSV PCR-positive pig at different within herd prevalence, or by conveniently selecting three ages of growing pigs (6-, 12- and 15 weeks of age). At each farm, recently dead or humanely euthanized pigs (i.e., <24 h) were included in the study. First, pigs were put on the ground at an area designated by the farm workers, managers, or veterinarians. Afterwards, the pigs were put on right lateral recumbency on a flat surface and, by using the proximal end of the ulna, also known as the olecranon, the area within the thoracic cavity where the heart lies would be identified. Once the punction area was identified, a sterile needle was introduced perpendicularly in-between the ribs and through the intercostal muscles to reach the heart (Figures 1, 2).

If a vacutainer system was used and blood would not immediately fill the collection tube, the thoracic cavity was compressed by placing the sampler hand on the thorax and with the index and middle fingers either side of the thoracic cavity lightly pressing the chest. In the case of the use of a syringe, aspiration was performed until 3–10 ml of blood was collected.

For newborn and suckling piglets, a 20G × 1 $\frac{1}{2}$” needle (BD Vacutainer^®^ PrecisionGlide™, Becton, Dickinson and Company, Franklin Lakes, NJ, USA) attached to a vacutainer tube holder (BD Vacutainer^®^ one-use holder, Becton, Dickinson and Company, Franklin Lakes, NJ, USA) was used. For growing pigs, blood collection was performed using the same puncture method used for suckling piglets but in this case a 16G × 3” needle (Air-Tite Products Co., Inc., Virginia Beach, VA, USA) attached to a 10 ml syringe was used. Once the blood sample was obtained, it was transferred to a sterile blood collection tube without anticoagulant (BD Vacutainer^®^, Becton, Dickinson and Company, NJ, USA). Samples were then submitted to the University of Minnesota diagnostic veterinary laboratory for RT-PCR testing for PRRSV as this pathogen was the main target of both of the studies.

A total of 286 CP blood samples were collected originating from 196 suckling and 90 growing pigs. In nine cases, sample collection did not yield enough serum volume for testing, and in four, the RT-PCR reaction was inhibited. Out of the remaining 273 samples, PRRSV was detected in 95% of the samples with a median [quartile (Q) 1, Q3] cycle threshold value of 21.5 (17.1, 28.5), a minimum of 10.8 and maximum of 35.5.

## Practical insights and broader applications for disease surveillance

Our results highlight the feasibility of obtaining blood samples from recently dead pigs of different ages through CP for viral disease (i.e., PRRSV) diagnosis and surveillance. Obtaining this sample was not only possible but welfare-friendly and avoided blood spillage and thus environmental contamination, supporting our efforts for disease containment. If attempting to collect this blood sample, certain aspects have to be considered. For instance, in our case the detection of PRRSV was not a definitive indication that this was the cause of death which indicates that finding a specific pathogen using this method should not be used as a definitive diagnosis. Another consideration is that the time between death and sampling can influence not only sample collection success but also pathogen detection. The size of the pig also needs to be taken into consideration, particularly regarding the collection materials utilized. Furthermore, non-veterinary personnel will require some training related to the anatomy of the thoracic cavity of the pig; however, we believe that this sampling technique can be quickly and effectively taught. While the data presented focused on PRRSV detection, it can be speculated that other viruses reaching the blood stream may also be detected in blood samples obtained from dead pigs. Lastly, this specimen should be considered as a complementary sampling tool, especially during outbreak investigations as CP is not intended to replace a full necropsy which is key for definitive diagnosis in pigs with clinical disease.

## Conclusion

Blood collection from dead pigs is a viable welfare-friendly alternative for PRRSV detection. After training to collect this specimen, CP is an alternative to obtaining a blood sample for surveillance and diagnosis of pathogens of interest (i.e., PRRSV, ASF, CSF) while minimizing blood spillage and environmental contamination which can increase the risk of pathogen dissemination.

## Data Availability

Additional data that support the findings of this study are available from the corresponding author upon reasonable request. Requests to access these datasets should be directed to corzo@umn.edu.
